# Long Noncoding RNA DICER1-AS1 Functions in Methylation Regulation on the Multi-Drugresistance of Osteosarcoma Cells *via* miR-34a-5p and GADD45A

**DOI:** 10.3389/fonc.2021.685881

**Published:** 2021-07-09

**Authors:** Feng Wang, Lingsuo Kong, Youguang Pu, Fengmei Chao, Chunbao Zang, Wei Qin, Fangfang Zhao, Shanbao Cai

**Affiliations:** ^1^ Department of Oncology, The First Affiliated Hospital of University of Science and Technology of China (USTC), Division of Life Sciences and Medicine, University of Science and Technology of China, Hefei, China; ^2^ Department of Anesthesiology, West district of The First Affiliated Hospital of USTC, Division of life Sciences and Medicine, University of Science and Technology of China, Hefei, China; ^3^ Department of Cancer Epigenetics Program, Anhui Provincial Cancer Hospital, Division of Life Sciences and Medicine, University of Science and Technology of China, Hefei, China; ^4^ Department of Radiation Oncology, Anhui Provincial Cancer Hospital, Division of Life Sciences and Medicine, University of Science and Technology of China, Hefei, China; ^5^ Department of Science and Education Section, Anhui Provincial Cancer Hospital, Division of Life Sciences and Medicine, University of Science and Technology of China, Hefei, China; ^6^ Department of Orthopedic Surgery, Anhui Provincial Cancer Hospital, Division of Life Sciences and Medicine, University of Science and Technology of China, Hefei, China

**Keywords:** osteosarcoma, drug resistance, DICER1-AS1, miR-34a-5p, GADD45A

## Abstract

Osteosarcoma (OS) is a common malignant bone tumor that commonly occurs in children and adolescents. Long noncoding RNAs (lncRNAs) are recognized as a novel class of regulators of gene expression associated with tumorigenesis. However, the effect and mechanism of lncRNAs in OS tumorigenesis and drug resistance have not been characterized. The purpose of the study is to screen potential biomarker and therapeutic target against OS. We compared the lncRNA expression profiles between OS cell lines with different drug resistance levels using RNA-seq analysis and found that lncRNA DICER1-AS1 was significantly differentially expressed in multi-drugresistant OS cells SJSA-1 versus multi-drugsensitive OS cells G-292. Bisulfite Sequencing PCR (BSP) assay was performed to analyze the differential methylation status of the promoter region of DICER1-AS1 in four OS cells. Subsequently, *in vitro* gain- and loss-of-function experiments demonstrated the roles of DICER1-AS1 and miR-34a-5p in the multi-drugresistance of OS cells. The main findings is that DICER1-AS1 directly binds to miR-34a-5p, and their expression has a negative correlation with each other. The hypermethylation of the promoter region of DICER1-AS1 silenced its expression in the drugresistant cells SJSA-1 and MNNG/HOS. Moreover, we found that growth arrest and DNA damage-inducible alpha (GADD45A) participates in the DICER1-AS1/miR-34a-5p-regulated drug resistance of OS cells, probably *via* the cell cycle/pRb-E2F pathway. Our results revealed DICER1-AS1/miR-34a-5p-regulated drug resistance of OS cells, a new lncRNA-regulated network in OS tumorigenesis, suggested that DICER1-AS1 can be considered as a potential biomarker and therapeutic target against OS cells.

## Introduction

Noncoding RNAs, especially microRNAs (miRNAs) and long noncoding RNAs (lncRNAs), are reported to participate in the proliferation, apoptosis, metastasis, invasion and drug resistance of tumors (1, 2). Increasing evidence has shown that miRNAs are related to oncogenes and tumor suppressor genes, as well as the biological behaviors of tumor cells, such as invasion, metastasis, proliferation, and apoptosis (3–5). LncRNAs are defined as a class of RNAs longer than 200nt without coding potential, although some lncRNAs can encode functional small peptides (6, 7). In recent years, an increasing number of studies have demonstrated that lncRNAs are key regulators of diverse biological processes (8). More importantly, lncRNAs play roles in regulating gene expression at different levels, including chromatin modification, transcriptional and posttranscriptional processing (9, 10). Notably, some lncRNAs are involved in various diseases, including cancers, leading to carcinogenesis and the development of cancer (11, 12). To date, distinct mechanisms have been proposed for lncRNA-regulated gene expression. For example, lncRNA HULC acts as a scaffold or guide to regulate interactions between proteins and genes (13), whereas lncRNA ROR acts as a decoy to bind to proteins or miRNAs (14). By contrast, some lncRNAs function as enhancers to modulate transcription of their targets after being transcribed from enhancer regions or their neighboring loci (15). In 2013, Karreth et al. reported a new lncRNA regulatory circuitry in which lncRNAs may function as competing endogenous RNAs (ceRNAs) and crosstalk with miRNAs by competitively binding to their common miRNAs (16). An increasing number of studies have demonstrated the functional roles of lncRNAs in OS tumorigenesis. For example, lncRNA FOXC2-AS1 and its antisense transcript FOXC2 form an RNA-RNA structure that regulates doxorubicin resistance in OS cells (17). LncRNA PVT1 is upregulated in OS cells and contributes to cell metastasis *via* the miR-497/HK2 pathway (18). These reports suggest the important roles of lncRNAs in OS tumorigenesis.

DNA methylation (methylation of 5’-carbon atom of cytosine ring in CpG island) epigenetically modifies gene expression and participates in regulating various cellular processes (19, 20). Abnormal DNA methylation in cancer cells is closely related to the formation of drug resistance in clinical chemotherapy (21, 22). Notably, Hundreds of cancer-related genes contain CpG islands, indicating that their transcription may be regulated by DNA methylation (23). Recent studies showed that hypermethylation of lncRNA C5orf66-AS1 promoter may serve as a potential prognostic marker in predicting gastric carcinoma patient survival (24). A previous study showed that doxorubicin and cisplatin could induce similar genomic methylation levels in breast cancer cell MCF-7, suggesting that abnormal DNA methylation may confer the drug resistance of breast cancer cells (17).

Osteosarcoma (OS) is a common malignant bone tumor that displays highly aggressive and early systemic metastasis (25). It remains the leading cause of mortality among children and adolescents (26). Drug resistance hampers the efficacy of clinical therapies for OS patients. Therefore, it is urgently needed to identify reliable biomarkers for early diagnosis and more efficient treatment of human OS. Increasing evidence has shown that the dysregulation of lncRNAs is associated with OS pathogenesis and drug resistance. For example, the downregulation of tumor suppressor lncRNA TUSC7 (tumor suppressor candidate 7, TUSC7) could promote OS cell proliferation *in vitro* (27). Notably, it was found that lncRNA ODRUL may reduce the sensitivity against doxorubicin in OS cells by inducing the expression of ABCB1, which is classically related to multi-drugresistance (28). LncRNA LINC00161 was shown to play an essential role in cisplatin-induced apoptosis and thus attenuates OS drug resistance (29). Despite the increasing number of studies, the mechanism underlying lncRNA-mediated OS drug resistance remains poorly understood. In the present study, we compared the lncRNA expression profiles between OS drugsensitive and drugresistant cell lines using RNA-seq analysis and found that lncRNA DICER1-AS1 was significantly differentially expressed in multi-drugresistant OS cells SJSA-1 versus multi-drugsensitive OS cells G-292. Subsequently, we showed that DICER1-AS1 plays a pivotal role in OS drug resistance by gain- and loss-of-function experiments. Mechanistically, we demonstrated that DICER1-AS1 interacts with miR-34a-5p and affects the expression of growth arrest and DNA damage-inducible alpha (GADD45A) for the regulation of apoptosis and cell cycle. Our results not only provide novel insights into the drug resistance of OS, but also offer hints for developing new biomarkers and therapeutic targets of OS.

## Materials and Methods

### Cell Lines and Culture Condition

Four osteosarcoma cancer cell lines—G-292 (CRL-1423), 143B (CRL-8303), SJSA-1 (CRL-2098) and MNNG/HOS (CRL-1547) were purchased from ATCC. Cells were added 10% fetal bovine serum (PAN biotechnology), 100 U/ml penicillin and 100 mg/ml streptomycin (WISTENC) to DMEM or RPMI1640 (biological industry) medium, and 5% CO_2_ was added to humidified air at 37°C.

### RNA Extraction and qRT-PCR Analyses

Total RNA was extracted from cultured cells with Trizol reagent (Tiangen biotechnology). For qRT-PCR, RNA was retrieved to the cDNA using a reverse transcription kit (Takara). In addition, the RNA levels of DICER1-AS1 and GADD45A genes were quantified by qRT-PCR analysis, and the TaqMan probes with different fluorescence intensity were used in the FTC-3000p PCR instrument (Funglyn Biotech). The level of beta-actin was normalized by 2^−ΔΔCt^ before comparing the relative levels of target genes. For miR-34a-5p expression detection, reverse transcription was performed according to the applied biological system TaqMan microRNA analysis protocol (Takara). The primer sequences are listed in **Supplementary Figure 1** of **Supplementary Materials**.

### RNA-Sequencing

SJSA-1 or MNNG/HOS cells were infected with pEZ-lv201.1-DICER1-AS1 or pEZ-lv201.1 before large-scale RNA sequencing. Total RNA was extracted by Trizol reagent, and DNA was produced by gene specific primers or random primers. Illumina-Hiseq 4000 system was used for RNA sequencing and Illumina-Hiseq 2000 system was used for library sequencing (BGI Technology Company). A single-ended library was prepared according to the Illumina-Truseq RNA sample preparation kit (Illumina) scheme, which has been described in our previous report (30).

### Fluorescence *In Situ* Hybridization

The cells were washed with PBS and fixed with 4% paraformaldehyde for 15 min at room temperature. Cells were permeated into PBS containing 0.5% Triton X-100 for 15 min at 4°C, and then washed in PBS for 5 min. After that, 70%, 85% and 100% ethanol were used to dehydrate for 3 min. The FISH probe was hybridized in a humid chamber at 75°C for 5 min to denaturate, and then the fluorescence *in situ* hybridization kit (Gene Pharma) was used overnight in the darkness at 37°C. The slides were washed three times with buffer F (20×SSC with 0.1% Tween-20). The slides were washed at room temperature for 5 min with 2×washing buffer C (40×SSC) and washing buffer C (20×SSC), respectively. The slides were dyed with DAPI for 20 min in darkness. The DICER1-AS1 FISH probe was designed and synthesized with Genemarma. 18S FISH probe were used as cytoplasm control. All images were obtained by fluorescence microscopy or confocal microscopy (Nikon).

### Cell Transfection

Using riboFECT CP transfection kit provided by Guangzhou Ribobio, China, miRNA mimic, inhibitor, short hairpin RNA (shRNA) or DNA plasmid transfection was carried out on 24-well plate. In the functional analysis of DICER1-AS1, 100 nM shRNA-DICER1-AS1 and 100 nM shRNA were introduced into the cells in the culture medium, and then harvested for further detection. In luciferase analysis, small RNA inhibitor (100 nM) or mimic (100 nM) and psicheck-2 (500 ng per pore) containing WT or mutated DICER1-AS1 sequence were introduced into cells. Cell harvesting was used for 48 h of double luciferase analysis. The mimic, inhibitor, shRNA sequences are listed in **Supplementary Figure 1** of **Supplementary Materials**.

### Drug Resistance Profiling (IC_50_ Measurements)

The clinical grade chemotherapeutic drugs used in this paper of are Etop (etoposide) supplied by Hengrui, Jiangsu, China, MTX (Methotrexate) supplied by Lingnan, Guangdong, China, CDDP (cisplatin) supplied by Haosen, Jiangsu, China, Carb (carboplatin) supplied by Qilu, Shandong, China and Dox (doxorubicin) supplied by Haizheng, Zhejiang, China.

Cells viability was measured using cell counting kit 8 (CCK-8) (Bimake). The IC_50_ values with the no-drug control as the reference were calculated. The relative drug resistance was presented as the fold change in the IC_50_ of the cell lines relative to the lowest IC_50_ (**Supplementary Figure 2**).

### Cell Apoptosis and Cell Cycle Analysis

Cells were diluted with 150 μl of 1×annexin-binding buffer, and then 5 μl of FITC-labeled enhanced-annexin V and 5 μl (20 μg/ml) of propidium iodide (PI) were added. Then the cells were incubated in the dark for 15 min at room temperature. Flow cytometry was conducted on a FACSCalibur instrument (FACSVerse).

Cells were fixed in 70% cold ethanol for 24 h at 4°C. Then were stained with 50 µg/ml PI at room temperature for 30 min in the dark. The cell cycle was evaluated and the results were analyzed.

### DNA Methylation Analysis

Genomic DNA was isolated by standard phenol/chloroform purification method, identified by 0.7% agarose gel electrophoresis, and then transformed into bisulfite by EZ-DNA methylation gold Kit (ZYMO research, USA). The bisulfite modified DNA was amplified by Qiagen HotStarTaq DNA polymerase and the amplified fragments were sequenced. The original sequence data file was processed, and the area ratio of C/C+T of primary CpG dinucleotides was calculated by the percentage of methylation, then the curve was drawn (31–33) (**Supplementary Figure 3**).

### Western Blot Analysis

Total cell solutes were dissolved in a solution buffer (60 mM Tris-HCl, pH 6.8, 2% SDS, 20% glycerol, 0.25% bromophenol blue and 1.25% 2-mercaptoethanol) and heated for 10 min at 95°C before electrophoresis. The proteins were separated by SDS-polyacrylamide gel electrophoresis (SDS-PAGE) and transferred to PVDF membrane (microporous). Immunoblotting was performed on the cell membrane with the first-order antibody. The AffiPuror goat was then incubated with IgG AffiPury or peroxidase coupled AffiPury goat to resist rabbit IgG. GAPDH was used as a control, which was normalized for the quantitation of target proteins. All the full-length unprocessed gels of immunoblots were provided in **Supplementary Figure 4** of **Supplementary Materials**.

### Luciferase Reporter Assay

Luciferase reporting is based on psiCHECK2 vector (Promega). In order to construct psiCHECK2-DICER1-AS1-WT or mut, a partial length sequence of DICER1-AS1-WT or mut containing a presumed miR-34a-5p binding site was synthesized and cloned into psiCHECK2 vector. Luciferase reporter was co-infected with miR-34a-5p mimic, miR-34a-5p-mut mimic, miR-34a-5p inhibitor, miR-34a-5p-mut inhibitor or NC in OS cells, according to manufacturer’s guidelines. Relative luciferase activity was measured by double Luciferase Report Analysis System (Promega) and Proega glomerular 20/20 photometer. As mentioned above, the activity of related luciferase was analyzed (5, 30).

### Statistical Analysis

The data are presented in the form of average, and the error bar indicates that S.D. Statistical analysis was carried out with graphpad prism 5 and Excel. Statistical analysis between groups was conducted by double-tailed t-test and one-way ANOVA, p<0.05 with statistical significance.

## Results

### DICER1-AS1 Is Involved in the Regulation of OS Cell Drug Resistance

**Figure 1:**
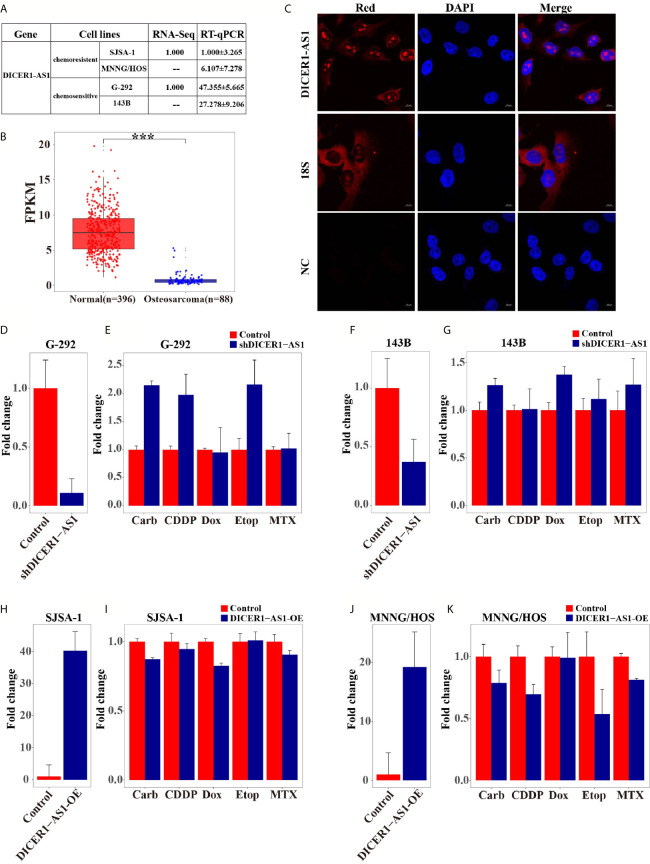
DICER1-AS1 is involved in the regulation of OS cells drug resistance. **(A)** The relative lncRNA-seq and real-time PCR analyse level (fold) of DICER1-AS1 in drugsensitive G-292 and 143B cells versus drugresistant SJSA-1 and MNNG/HOS cells. **(B)** Relative expression levels of the DICER1-AS1 FPKM data in 88 cases osteosarcoma cancer samples and 396 cases surrounding normal tissues. **(C)** RNA fluorescence in situ hybridization showing the localization of DICER1-AS1 in G-292 cells. Cells were incubated with DICER1-AS1 sense probes (5' GCCCA+A+CGCCA+AGGTCCA+GTCCA 3'). After DAPI staining, fluorescence was observed under a fluorescence microscope. Scale bar, 25μm. **(D, F)** The relative DICER1-AS1 expression level (fold) in G-292(D) and 143B(F) cells transfected with shDICER1-AS1 versus the negative control(NC). **(E, G)** CCK8 assay showing cell death triggered by an IC_50_ dose of drug in G-292 **(E)** and 143B **(G)** cells transfected with shDICER1-AS1 *versus* the negative control(NC) assayed 72 h after treatment with the IC_50_ dose of drugs. **(H, J)** The relative DICER1-AS1 expression level (fold) in SJSA-1(H) and MNNG/HOS(J) cells infected with DICER1-AS1-OE versus the negative control (NC). **(I, K)** CCK8 assay showing cell death triggered by an IC50 dose of drug in SJSA-1(I) and MNNG/HOS(K) cells infected with DICER1-AS1-OE *versus* the negative control(NC) assayed 72 h after treatment with the IC_50_ dose of drugs. The data are mean±SD of three separate experiments. “-” indicates no detection in the array analysis. ***p value < 0.001.

We have previously identified that miR-34a-5p is involved in the drug resistance of OS cells (5, 30, 34). To further investigate the underlying mechanism of OS drug resistance, we performed lncRNA-seq and screened the differentially expressed lncRNAs in multi-drugresistant SJSA-1 cells versus multi-drugsensitive G-292 cells (GEO accession number: GSE153786), putative lncRNAs expressed higher in G-292 cells were identified (**Supplementary Figure 5A**). Among the putative lncRNAs, we predicted the potential miR-34a-5p targets using the following online databases: targetScan, miRanda and picTar, which might be negatively correlated with the expression of miR-34a-5p (**Supplementary Figure 6**), twelve lncRNAs (such as LRP4-AS1, C21orf90, DICER1-AS1 et. al.) were the proposed as the targets of miR-34a-5p, as demonstrated in the hierarchical clustering profiling (**Supplementary Figure 5B**). We further verified the expression of these 12 lncRNAs and confirmed 7 of them, including LRP4-AS1, DICER1-AS1, TERC, ANK3, LINC00693, TTN-AS1 and CRHR1-IT1. Among them, DICER1-AS1 had the most significant difference in expression between SJSA-1 and G-292 cells (**Figure 1A**). Next, we examined the expression of DICER1-AS1 in osteosarcoma cancer sample, in TCGA and target database, osteosarcoma samples have neither with differentiation of drug resistance with drug sensitivity, nor normal control data, so we choose the surrounding normal tissues as the control, we extract their FPKM data and make comparison, the results revealed that it was significantly downregulated in osteosarcoma cancer samples and then surrounding normal tissues (**Figure 1B**, **Supplementary Figure 3**). RNA fluorescence *in situ* hybridization results showed that DICER1-AS1 was localized in both the cytoplasm and nucleus, with nucleus localization being predominant (**Figure 1C**, **Supplementary Figure 4**). Thereafter, we compared the expression profile of DICER1-AS1 in multi-drugresistant OS cells (SJSA-1 and MNNG/HOS) and multi-drugsensitive OS cells (G-292 and 143B) by lncRNA-seq and qRT-PCR analysis. The results demonstrated the lower expression of DICER1-AS1 in multi-drugresistant OS cells compared with that in the multi-drugsensitive OS cells (**Figure 1A**), indicating that DICER1-AS1 might function in the multi-drugsensitivity of OS.

To further verify that DICER1-AS1 is involved in the regulation of OS drug resistance, we mandated to reverse the expression of DICER1-AS1 in OS cells. We knocked down the expression of DICER1-AS1 by transfection with shDICER1-AS1 in either G-292 or 143B cells, the multi-drugsensitive OS cells (**Figures 1D, F**). Downregulation of DICER1-AS1 increased the drug-resistance ability, as revealed by the relative cell survival, against the following drugs: etoposide (Etop), cisplatin (CDDP), and carboplatin (Carb) in G-292 cells (**Figure 1E**), and etoposide (Etop), methotrexate (MTX), carboplatin (Carb) and doxorubicin (Dox) in 143B cells (**Figure 1G**). On the contrary, when we upregulated the DICER1-AS1 level by lentivirus-infection of DICER1-AS1 in SJSA-1 or MNNG/HOS cells, the multi-drugresistant OS cells, as revealed by real-time PCR (**Figures 1H, J**), the drug-resistance capability against Carb and Dox was reduced in SJSA-1 cell (**Figure 1I**) and this is also the case in MNNG/HOS cell except for Dox (**Figure 1K**). These results showed that DICER1-AS1 indeed involves in the inhibition of the multi-drugresistance of OS cells.

### DICER1-AS1 Hypermethylation in Tumor Tissues and the Multi-Drugresistance of OS Cells

**Figure 2:**
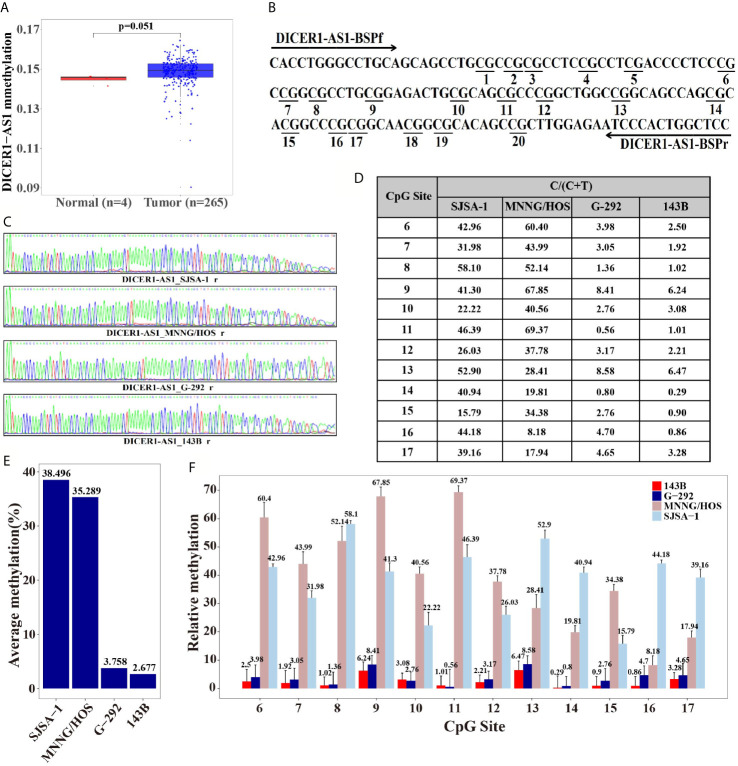
Differential methylation of the DICER1-AS1 gene in SJSA-1, MNNG/HOS, G-292 and 143B cells. **(A)** The methylation of DICER1-AS1was upregulated in tumor (TCGA data, red dot for tumor tissue, n=265; blue dot for normal tissue, n=4). **(B)** The CpG dinucleotides and the BSP primers of the DICER1-AS1 gene are shown. **(C)** The original reverse sequencing results of the bisulfite-converted DNA of the four OS cells are shown. **(D–F)** The percentage of CpG methylation of the four OS cells was summarized in the plot.

To further elucidate the regulation mechanism of DICER1-AS1, we analyzed the DICER1 gene in the UCSC database. The physical location of the DICER1 coding gene overlaps with DICER1-AS1, and the methylated DICER1 was reported to be involved in endometrial carcinoma invasion (35). Meanwhile, DICER1-AS1 is rich in CpG islands as analyzed by the UCSC database (**Supplementary Figure 5B**), suggesting that DICER1-AS1 may be associated with methylation. Therefore, we first compared the DNA methylation levels of DICER1-AS1 in the tumor and normal tissues *via* the Cancer Genome Atlas (TCGA) datasets (http://cancergenome.nih.gov) (**Figure 2A**). The results showed that DICER1-AS1 in the tumor tissues has a general hypermethylation status, as compared to the normal tissues.

The differential methylation status of the promoter sequence of DICER1-AS1 was analyzed in four OS cells by the Bisulfite Sequencing PCR (BSP) assay. The analyzed sequence contained 20 CG points (**Figure 2B**), among which the sixth to seventeenth points were considered valid because of the instability of the peaks on both ends during sequencing (**Figures 2C, D**). The average methylation ratios of the DICER1-AS1 gene in SJSA-1 and MNNG/HOS were 38.5% and 35.3%, respectively, much higher than those in G-292 and 143B cells (**Figures 2E, F** and **Supplementary Figure 5**). The methylation degree was negatively correlated with its expression value, strongly indicating that the expression of DICER1-AS1 might be conducted by dual regulation *via* DNA methylation and miR-34a-5p.

### DICER1-AS1 Promotes OS Cells Apoptosis and G2/M Arrest

**Figure 3:**
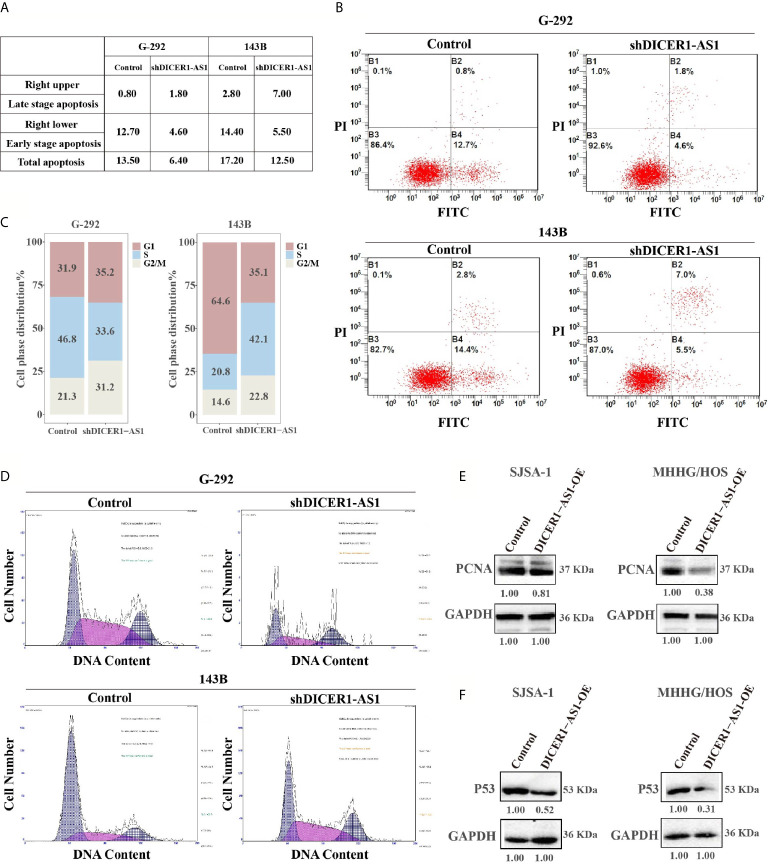
DICER1-AS1 is involved in OS cells apoptosis and G2/M arrest. **(A, B)** The effects of the forced reversal of DICER1-AS1 level on the apoptosis of G-292 and 143B cells by FACS analysis in plot and in the original with a graph of the analyzed data and plots of the original FACS data. **(C, D)** The effects of the forced reversal of DICER1-AS1 level on the cell cycle distribution of G-292 and 143B cells by FACS analysis in plot and in the original. **(E)** The protein levels of cell apoptosis related gene PCNA in SJSA-1 and MNNG/HOS cells infected with DICER1-AS1-OE versus the negative control(NC) determined by western blot analyses. **(F)** The protein levels of cell cycle related gene p53 in SJSA-1 and MNNG/HOS cells infected with DICER1-AS1-OE versus the negative control(NC) determined by western blot analyses.

We performed both gain- and loss-of-function studies to investigate the pathological role of DICER1-AS1 in OS cells. The forced knockdown of the DICER1-AS1 level decreased the percentage of apoptotic cells from 13.50% to 6.40% in G-292, and 17.20% to 12.50% in 143B cells, respectively (**Figures 3A, B**). Furthermore, FACS analysis showed that knockdown of DICER1-AS1 increased the cell quantity in the G2/M phase, thus inhibiting DNA replication in G-292 and 143B cells (**Figures 3C, D**). These results imply that DICER1-AS1 could play a role in arresting cells in the G2/M phase to facilitate the necessary repair of cellular damage induced by stress, such as anti-cancer drugs.

Generally, overexpression of DICER1-AS1 might down regulate the expression of genes in the apoptosis-related signaling pathway. We found that PCNA was down regulated following the overexpression of DICER1-AS1 in both SJSA-1 and MNNG/HOS cells (**Figure 3E**). Additionally, following the overexpression of DICER1-AS1, p53 expression was drastically down regulated in SJSA-1 and MNNG/HOS cells (**Figure 3F**). The results clearly suggest that DICER1-AS1 is involved in the regulation of the cell cycle and apoptosis pathway.

### DICER1-AS1 Regulates the Expression of Growth Arrest and DNA Damage-Inducible Alpha (GADD45A)

**Figure 4:**
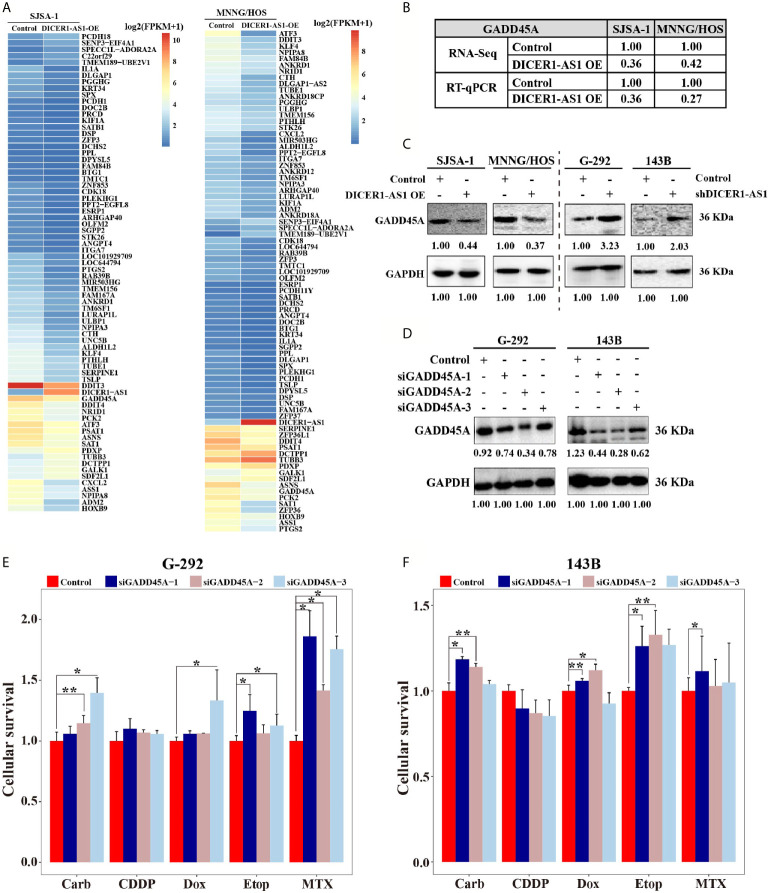
DICER1-AS1 interacts with growth arrest and DNA damage inducible alpha (GADD45A) in OS cells. **(A)** Hierarchical clustering analysis of mRNAs that were differentially expressed between DICER1-AS1-overexpression with control vector in SJSA-1 and MNNG/HOS cells respectively. **(B)** The relative GADD45A levels (fold) of DICER1-AS1-overexpression compared with control vectors measured by both RNA-seq and qRT-PCR analyses in MNNG/HOS and SJSA-1 cells, respectively. **(C)** The GADD45A protein levels with DICER1-AS1-overexpression compared with control vector (pEZ-lv201.1) in SJSA-1 and MNNG/HOS cells, respectively, and in the shDICER1-AS1-transfected versus the sh-NC-transfected G-292 and 143B cells, respectively. **(D)** The levels of GADD45A protein levels determined by western in the three different region’ siRNAs transfected into G-292 and 143B cells versus the NC-transfected cells, respectively. **(E, F)** The CCK8 assays showing cell death triggered by an IC_50_ dose of drug in G-292 and 143B cells transfected with GADD45A three different siRNAs versus the negative control (NC) assayed 72 h after treatment with the IC_50_ dose of drugs. *p value < 0.05; **p value < 0.01.

To further identify the target genes of DICER1-AS1, we first detected the activities of seventeen signaling pathways with overexpressed DICER1-AS1 in SJSA-1 and MNNG/HOS cells. In the two independent experiments, only the cell cycle/pRb-E2F pathway showed similar changing trends in both SJSA-1 and MNNG/HOS cells (**Table 1**). Next, we profiled mRNA-seqs that were differentially expressed between DICER1-AS1-overexpression and control vector in SJSA-1 and MNNG/HOS cells, and got the GEO accession number of GSE153787 (**Figure 4A**). The results showed that dozens of genes were differentially expressed in both cells. Among these genes, we screened the mRNAs that might participate in the cell cycle/pRb-E2F pathway in both SJSA-1 and MNNG/HOS cells (**Supplementary Figure 6**). The results showed that growth arrest and DNA damage-inducible alpha (GADD45A) expression was significantly downregulated in both SJSA-1 and MNNG/HOS cells (**Table 2**, **Supplementary Figure 7**), as further confirmed by qRT-PCR assays (**Figure 4B**).

**Table 1:** The activities of seventeen signaling pathways with overexpressed DICER1-AS1 in SJSA-1 and MNNG/HOS cells.

Pathway	Transcription Factor	SJSA-1		MNNG/HOS	
		DICER1-AS1-O/E/NC		DICER1-AS1-O/E/NC-O/E	
Wnt	TCF/LEF	0.104	5.272	15.023	0.897
Notch	RBP-Jκ	0.086	3.736	0.278	1.190
p53/DNA Damage	p53	1.179	0.926	0.513	0.688
TGFβ	SMAD2/3/4	0.271	1.747	2.346	1.273
Cell cycle/pRb-E2F	E2F/DP1	0.331	0.219	0.426	0.736
NFκB	NFκB	0.175	8.798	12.259	0.721
Myc/Max	Myc/Max	0.617	1.063	6.563	1.522
Hypoxia	HIF1A	1.036	3.257	3.998	0.849
MAPK/ERK	Elk-1/SRF	0.384	6.966	12.575	0.750
MAPK/JNK	AP-1	1.229	11.438	3.325	0.885
ATF2/ATF3/ATF4	ATF2/ATF3/ATF4	0.868	7.421	2.161	0.480
cAMP/PKA	CREB	1.422	2.336	0.378	0.799
MEF2	MEF2	1.554	5.136	1.188	0.594
Hedgehog	GLI	1.534	3.494	0.869	1.223
PI3K/AKT	FOXO	1.532	1.984	497.609	1.282
IL-6	STAT3	1.096	0.744	26.098	1.063
PKC/Ca++	NFAT	0.558	2.206	2.030	1.145
Negative Control		1.000	1.000	1.000	1.000

**Table 2:** GADD45A expression in SJSA-1 and MNNG/HOS cells.

MNNG/HOS		SJSA-1	
Symbol	DICER – AS1 OE/Control	Symbol	DICER – AS1 OE/Control
FOSB	0.00337	PRH1	0.00699
FOS	0.00356	LOC100289561	0.01449
KRTAP4-8	0.00775	ALX1	0.02128
CHML	0.01087	LTB4R2	0.02128
NPIPB4	0.01235	SULT1A4	0.02222
FAM209B	0.01351	GYPC	0.02439
AMT	0.01639	SPRR2D	0.03030
NPHP3-ACAD11	0.01724	TRIM6-TRIM34	0.03571
KRBOX4	0.42262	CXCL5	0.34169
GRK4	0.42268	TUBE1	0.34203
MAP1LC3C	0.42291	LINC00592	0.34280
GADD45A	0.42294	ACBD7-DCLRE1CP1	0.34314
FBLN5	0.42308	SLC12A7	0.35623
SLFN13	0.42331	FAM231A	0.35694
LYST	0.42347	BIK	0.35714
RNF103	0.42347	GADD45A	0.35728
RASSF8	0.42350	IFI16	0.35770
ZNF641	0.42353	ETV7	0.35940
NA	0.42379	NCOA7	0.35963
HLTF	0.42380	FSBP	0.34831
FAM155A	0.42394	RAC2	0.34961
STAU2	0.42424	GAS5	0.34974
GIN1	0.42424	IL15RA	0.34990
TOPORS	0.42434	RIPK4	0.35000
TMTC3	0.42446	SATB1	0.35135
TBC1D3L	0.42453	LOC155060	0.35211
CPEB4	0.42456	DMRTA1	0.35345
NBPF20	0.42468	ITPR1	0.35358
RASD1	0.42484	DGAT2	0.35385
FBXO11	0.42497	IL12A	0.35394
ANKIB1	0.42516	SESN2	0.35451
TP53INP1	0.20870	SFMBT2	0.35484

To test whether GADD45A is indeed regulated by DICER1-AS1, we detected the protein level of GADD45A in different OS cells. With DICER1-AS1-overexpression in SJSA-1 and MNNG/HOS cells, the GADD45A level was significantly downregulated. By contrast, downregulation of DICER1-AS1 in G-292 and 143B cells upregulates the expression of GADD45A (**Figure 4C**). These results clearly demonstrated that GADD45A negatively correlates with the DICER1-AS1 level and might be a target of DICER1-AS1.

To test the role of GADD45A in OS drug resistance, we performed drug-resistance profiling with transfection of si-GADD45A in both G-292 and 143B cells. Consistently, transfection of each of the three siRNAs that are complementary with the different regions of GADD45A in both G-292 and 143B cells to downregulate GADD45A expression (**Figure 4D**). Downregulation of GADD45A by three different si-GADD45A increased the drug resistance for four drugs except CDDP in G-292 cell, indicating a higher cell survival rate (**Figure 4E**). Similar results were also found in 143B cell despite of the discrepancy of CDDP (**Figure 4F**), the similar trend of the IC_50_ changes in G-292 cells transfected with three different si-GADD45A (**Supplementary Figure 8**). The results suggest that GADD45A is involved in the drug resistance of OS cells.

### DICER1-AS1 Interacts With miR-34a-5p Through Direct Binding

**Figure 5:**
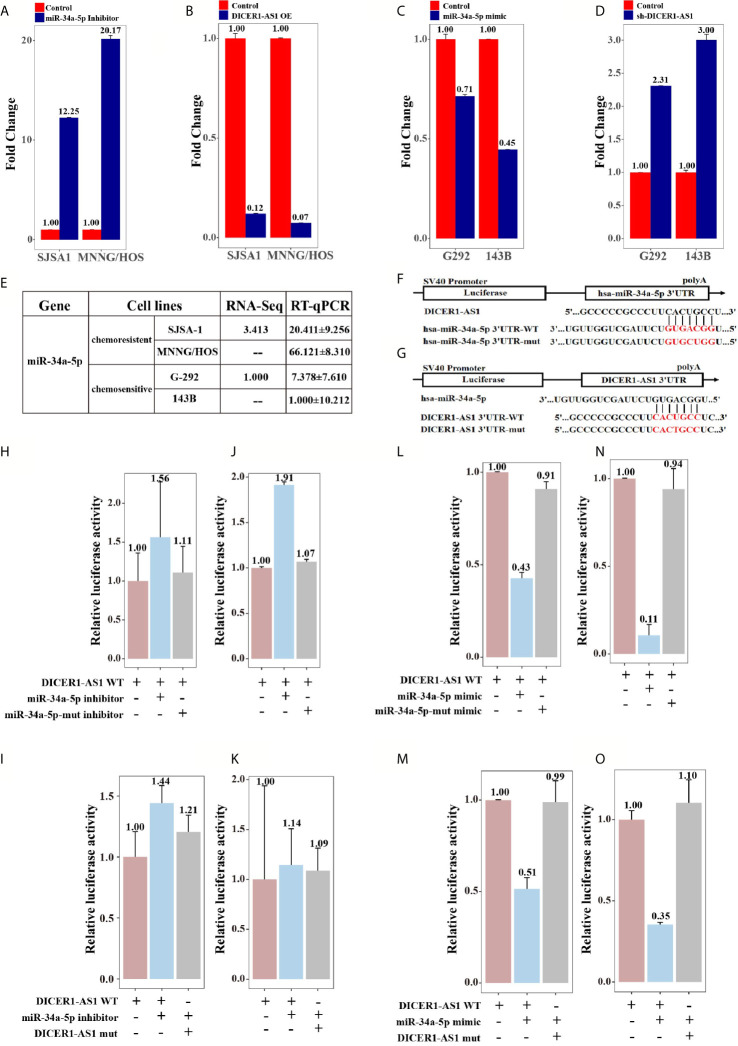
DICER1-AS1 interacts with miR-34a-5p through direct binding in vitro experiments. **(A)** The relative DICER1-AS1 expression level in the miR-34a-5p inhibitor-transfected SJSA-1 and MNNG/HOS cells versus the negative control (NC), as determined by qRT-PCR analyses. **(B)** The relative miR-34a-5p expression level in SJSA-1 and MNNG/HOS cells infected with DICER1-AS1-OE versus the negative control (NC). **(C)** The relative DICER1-AS1 expression level in the miR-34a-5p mimic-transfected G-292 and 143B cells versus the negative control (NC). **(D)** The relative miR-34a-5p expression level in G-292 and 143B cells transfected with shDICER1-AS1 versus the negative control (NC). **(E)** The relative miR-34a-5p level (fold) in G-292 and 143B cells versus SJSA-1 and MNNG/HOS cells measured by both miR-omic and qRT-PCR analyses were shown, “—” indicates no detection in the omic analysis. **(F)** Luciferase reporter constructs: WT and mut miR-34a-5p in the DICER1-AS1-binding sites were inserted into psiCHECK-2 vector. The red base region is the binding site. **(G)** Luciferase reporter constructs: WT and mut DICER1-AS1 in the miR-34a-5p-binding sites were inserted into psiCHECK-2 vector. The red base region is the binding site. **(H, J)** The relative luciferase activity of psiCHECK-2 containing WT-DICER1-AS1 co-transfected with NC, miR-34a-5p-inhibitor or miR-34a-5p-mut inhibitor in SJSA-1 and MNNG/HOS cells. **(I, K)** The relative luciferase activity of psiCHECK-2 containing WT or mutated DICER1-AS1 co-transfected with miR-34a-5p-inhibitor in SJSA-1 and MNNG/HOS cells. **(L, N)** The relative luciferase activity of psiCHECK-2 containing WT DICER1-AS1 co-transfected with NC, miR-34a-5p mimic or miR-34a-5p mut mimic in G-292 and 143B cells. **(M, O)** The relative luciferase activity of psiCHECK-2 containing WT or DICER1-AS1-mut co-transfected with miR-34a-5p-mimic in G-292 and 143B cells.

**Figure 6:**
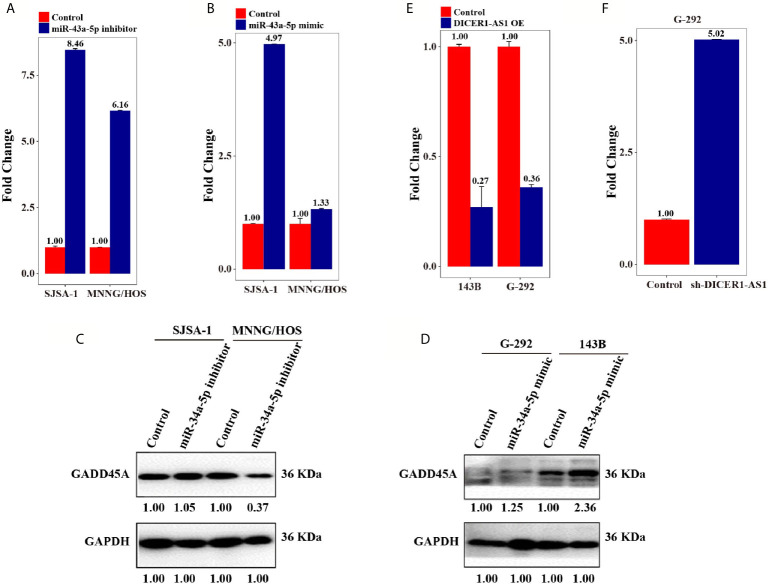
DICER1-AS1/miR-34a-5p/GADD45A involved in Cell cycle/pRb-E2F signaling pathway. **(A)** GADD45A mRNA level in the miR-34a-5p inhibitor-transfected SJSA-1 and MNNG/HOS cells versus the negative control (NC), as determined by qRT-PCR analyses. **(B)** GADD45A mRNA level in the miR-34a-5p mimic-transfected G-292 and 143B cells *versus* the negative control (NC). **(C)** The relative GADD45A expression level in SJSA-1 and MNNG/HOS cells infected with miR-34a-5p inhibitor versus the negative control (NC). **(D)** The relative GADD45A expression level in G-292 and 143B cells infected with miR-34a-5p mimics versus the negative control (NC). **(E)** The relative GADD45A expression level in SJSA-1 and MNNG/HOS cells infected with DICER1-AS1-OE *versus* the negative control (NC-OE). **(F)** The relative GADD45A expression level in G-292 cells infected with shDICER1-AS1versus the negative control (NC-OE).

To further investigate whether DICER1-AS1 is the target of miR-34a-5p, we reversely changed the expression level of DICER1-AS1 and miR-34a-5p. As shown in **Figure 6**, transfection of an inhibitor against miR-34a-5p into SJSA-1 or MNNG/HOS cells increased the expression levels of DICER1-AS1 to 12.25 or 20.17 folds, respectively (**Figure 5A**). Alternatively, we overexpressed DICER1-AS1 by transfection with DICER1-AS1-OE and found that the expression of miR-34a-5p was significantly reduced (**Figure 5B**). A similar effect of the expression profile of DICER1-AS1 and miR-34a-5p was also found in G-292 and 143B cells (**Figures 5C, D**). The results suggest that miR-34a-5p and DICER1-AS1 antagonize each other.

Previously, we reported the results of miR-34a-5p from miR-omic analysis of the G-292 and SJSA-1 cell lines and qRT-PCR analysis in SJSA-1, MNNG/HOS, G-292 and 143B cells and found that miR-34a-5p is upregulated in SJSA-1 and MNNG/HOS cells but is downregulated in G-292 and 143B cells (30, 34). Thus, the expression of miR-34a-5p is negatively correlated with that of DICER1-AS1 (**Figures 1A**, **5E**).

To examine if miR-34a-5p directly binds to DICER1-AS1, we performed a luciferase screening. First, we predicted the interaction region of DICER1-AS1 with miR-34a-5p using online databases. Next, we constructed luciferase reporter assays using the psiCHECK-2 vector with the insertion of wild-type or mutant DICER1-AS1 in the miR-34a-5p-binding sites (**Figures 5F, G**). The results indicated that miR-34a-5p might target DICER1-AS1 and down regulates DICER1-AS1. To further prove this, we systematically tested the miR-34a-5p-mediated effect of DICER1-AS1 expression using luciferase reporter assays. Cotransfection of DICER1-AS1 and the miR-34a-5p inhibitor into either SJSA-1 or MNNG/HOS cells significantly increased the luciferase activity, indicating an elevated expression of DICER1-AS1 following inhibition of miR-34a-5p (**Figures 5H, J**). By contrast, cotransfection of DICER1-AS1 and the miR-34a-5p-mut inhibitor showed almost no effect compared to that in the control cells. Similarly, when we mutated DICER1-AS1, no drastic differences were found even with cotransfection of the miR-34a-5p inhibitor (**Figures 5I, K**). Moreover, cotransfection of DICER1-AS1 and the miR-34a-5p mimic significantly downregulated the expression of DICER1-AS1 in G-292 and 143B cells, whereas either the DICER1-AS1 or miR-34a-5p mutant diminished this effect (**Figures 5L–O**). These results clearly suggest that DICER1-AS1 can interact with miR-34a-5p through direct binding.

### Regulatory Network of DICER1-AS1 in OS

The above results (**Figures 6A–D**) suggest that miR-34a-5p and DICER1-AS1 antagonize each other. Next, we detected the RNA and protein expression level of GADD45A accompanied by changing the level of either miR-34a-5p or DICER1-AS1. Following downregulation of miR-34a-5p in SJSA-1 and MNNG/HOS cells, the RNA expression level of GADD45A was upregulated (**Figure 6A**). However, following upregulation of miR-34a-5p in G-292 and 143B cells, the RNA expression level of GADD45A was also upregulated, although to a less extent (**Figure 6B**). With the transfection of miR-34a-5p inhibitor, where the miR-34a-5p will be downregulated, the GADD45A protein level was downregulated in MNNG/HOS cell, but showed a minor change in SJSA-1 cell (**Figure 6C**). Similarly, upregulation of miR-34a-5p in G-292 and 143B cells upregulated the GADD45A protein level (**Figure 6D**). The results suggest no direct correction between miR-34a-5p and GADD45A, similar results were confirmed by informatics analysis using predicted target genes websites of Targetscan, miRDB and microRNA.org. On the other hand, overexpression of DICER1-AS1 in SJSA-1 and MNNG/HOS cells resulted in reduced expression of GADD45A (**Figure 6E**), whereas downregulation of DICER1-AS1 in G-292 cells upregulated the expression of GADD45A (**Figure 6F**). The results revealed that the GADD45A level is negatively correlated with DICER1-AS1.

Considering that GADD45A is involved in cell cycle and apoptosis, we checked the expression of several pivotal proteins involved in these pathways (**Supplementary Figures 9** and **10**). As showed in **Figures 3E, F** following the overexpression of DICER1-AS1, the expression of p53 and PCNA was drastically downregulated in both SJSA-1 and MNNG/HOS cells. The results clearly suggest that DICER1-AS1 is indeed involved in the regulation of the cell cycle and apoptosis pathway. In summary, these results confirmed the regulatory network of DICER1-AS1/miR-34a-5p/GADD45A in OS tumorigenicity.

## Discussion

Cancer chemotherapy tolerance is a complex pathological process involving genetic and epigenetic changes of multiple genes (36, 37). Abnormal DNA methylation in cancer cells has been found to be closely related to the formation of drug resistance (38). Cancer cells suffer from a wide range of the epigenetic defects that are more tightly linked to the cancerous phenotypes.

DNA methylation is the best-characterized epigenetic mechanism in the regulation of gene expression, genomic imprinting, genome stabilization, and chromatin modification. The hypermethylated state of the promoter and enhancer regions tightly correlates with the transcriptionally silenced state of genes. Therefore, detection of the DNA methylation state of the promoter regions in patient samples promises a better way for both early detection and rationale personalized therapy of cancer. Notably, the aberrant DNA methylation has been reported to be involved in cancers. Recent studies have shown that expression alterations of lncRNA-encoding genes mediated by methylation can subsequently affect their downstream targets. Promoter hypermethylation of the lncRNA PLUT is predictive in patients with early stage I adenocarcinoma at high risk for early recurrence (39).

Moreover, recent studies have demonstrated that lncRNAs act as pivotal regulators of OS tumorigenicity because they participate in the pathogenesis process of OS, including cell growth, proliferation, invasion, migration, metastasis and cell prognosis (40–44). In this study, we found a lncRNA, DICER1-AS1, which negatively correlates with the expression of miR-34a-5p, a well-known regulator in OS drug resistance (5, 31, 35). Through gain- and loss-of function assays, we found that DICER1-AS1 suppresses the tumorigenicity of OS cells. Though, Dox may not affect cell survival in G-292 and MNNG/HOS cells drug resistance (**Figures 1G, J**). The discrepancy may be caused by different conditions and materials used in cell and animal experiments.

Similarly, our previous results suggested that miR-34a-5p plays roles in OS tumorigenicity, indicating the complicated network for noncoding RNA-regulated OS biology. Further *in vivo* and *in vitro* investigations revealed a central regulatory network of miR-34a-5p/DICER1-AS1/GADD45A. In detail, DICER1-AS1 and miR-34a-5p directly bind to each other and synergistically regulate OS drug resistance. Notably, a previous report found another miRNA, miR-30b, acts as a target of DICER1-AS1 to synergistically regulate OS proliferation, invasion and autophagy (44). Another report found the expression level of DICER1-AS1 in osteosarcoma tissues were significantly higher than those in corresponding noncancerous bone tissues, higher DICER1-AS1 had significant association with clinical stage and distant metastasis (45). This further suggests a complicated regulatory network for lncRNA-regulated cancer tumorigenicity. We further identified that GADD45A is one of the targets of DICER1-AS1 using mRNA profiling. These results enable us to propose a complicated regulatory pathway of OS drug resistance that involves DICER1-AS1, miR-34a-5p and GADD45A.

GADD45A, a ubiquitously expressed and DNA damage-responsive protein, a p53-regulated and DNA damage-inducible gene, is implicated in the protection against tumor malignancy. It plays important roles in suppressing cell proliferation, mediating cell cycle arrest, promoting apoptosis, inducing DNA repair, and stabilizing genomics (46–48). In accordance with previous reports, we found that up- or downregulation of DICER1-AS1 indeed altered the expression of the p53-regulated pathway. On the other hand, it is noteworthy that GADD45A promotes DNA demethylation through thymine DNA glycosylase (49–51), which correlates with the hypermethylation of DICER1-AS1 in drug-resistant OS cells. These results indicated a sophisticated feed-back regulation of GADD45A in OS drug resistance. The detailed mechanism underlying the biological functions of GADD45A involved in OS drug resistance remains to be elucidated in the near future.

Taken together, our findings in this work establish that targeting the miR-34a-5p/DICER1-AS1/GADD45A axis is a potential clinical strategy for therapeutic intervention in OS drug resistance. Our findings further support the notion of miR-34a-5p as a prospective drug to treat OS. Additionally, these results showed great promise for developing feasible diagnostic or prognostic biomarkers and prospective therapeutic targets based on DICER1-AS1. Further investigation and identification are still needed to elucidate the detailed mechanism of DICER1-AS1-mediated OS tumorigenesis.

In conclusion, our study demonstrated that DICER1-AS1 interacts with miR-34a-5p and targets GADD45A for the regulation of the cell cycle and apoptosis. Our results not only provide novel insights into the drug resistance of OS, but also offer hints for developing new biomarkers and therapeutic targets in OS.

## Conclusions

Our study demonstrated that DICER1-AS1 interacts with miR-34a-5p and controlling GADD45A for the regulation of the cell cycle and apoptosis. Our results not only provide novel insights into the drug resistance of OS, but also offer hints for developing new biomarkers and therapeutic targets in OS.

## Data Availability Statement

The datasets presented in this study can be found in online repositories. The names of the repository/repositories and accession number(s) can be found below: GEO database and the accession number is GSE153786.

## Author Contributions

Conception and design: FW and SC. Acquisition of data: FZ, WQ, FC, CZ, and LK. Analysis and interpretation of data: FW and LK. Writing, review, and/or revision of the manuscript: FZ and SC. All authors contributed to the article and approved the submitted version.

## Funding

This work was supported by the Fundamental Research Funds for the Central Universities granted to FZ (WK9110000008), LK (WK9110000090), and CZ (WK9110000132), respectively. The Youth Fund of Anhui Cancer Hospital granted to FZ and LK, respectively. The Youth Technical Backbone Fund of West Branch of the First Affiliated Hospital of USTC granted to CZ and LK, respectively.

## Conflict of Interest

The authors declare that the research was conducted in the absence of any commercial or financial relationships that could be construed as a potential conflict of interest.

## Abbreviations

OS, Osteosarcoma; lncRNA, long noncoding RNA; GADD45A, growth arrest and DNA damage inducible alpha; miR, microRNA; UTR, untranslated region; WT, wild-type vector; mut, mutant-type vector; PBS, phosphate-buffered saline; Dox, Doxorubicin; Etop, Etoposide; CDDP, Cisplatin; Carb, Carboplatin; MTX, Methotrexate; PI, propidium iodide; BSP, Bisulfite Sequencing PCR; CpG, Cytosine-phosphate-guanine.

## References

[1] Rasmussen MH Lyskjaer I Jersie–Christensen RR Tarpgaard LS Primdal–Bengtson B Nielsen MM. miR-625-3p Regulates Oxaliplatin Resistance by Targeting MAP2K6-p38 Signalling in Human Colorectal Adenocarcinoma Cells. Nat Commun (2016) 7:12436. 10.1038/ncomms12436 27526785 PMC4990699

[2] Koirala P Huang J Ho TT Wu F Ding X Mo YY. Lncrna AK023948 is a Positive Regulator of AKT. Nat Commun (2017) 8:14422. 10.1038/ncomms14422 28176758 PMC5309785

[3] Bartel DP. MicroRNAs: Target Recognition and Regulatory Functions. Cell (2009) 136:215. 10.1016/j.cell.2009.01.002 19167326 PMC3794896

[4] Pu Y Yi Q Zhao F Wang h Cai W Cai S. MiR-20a-5p Represses Multi-Drug Resistance in Osteosarcoma by Targeting the KIF26B Gene. Cancer Cell Int (2016) 16:64. 10.1186/s12935-016-0340-3 27499703 PMC4974744

[5] Pu Y Zhao F Li Y Cui M Wang H Meng X. The miR-34a-5p Promotes the Multi-Chemoresistance of Osteosarcoma Via Repression of the AGTR1 Gene. BMC Cancer (2017) 17:45. 10.1186/s12885-016-3002-x 28073349 PMC5223322

[6] Mattick JS Rinn JL. Discovery and Annotation of Long Noncoding RNAs. Nat Struct Mol Biol (2015) 22:5. 10.1038/nsmb.2942 25565026

[7] Guo W Dong Z Shi Y Liu S Liang J Guo Y. Aberrant Methylation-Mediated Downregulation of Long Noncoding RNA LOC100130476 Correlates With Malignant Progression of Esophageal Squamous Cell Carcinoma. Digestive Liver Dis (2016) 48:961. 10.1016/j.dld.2016.05.010 27338851

[8] Cech TR Steitz JA. The Noncoding RNA Revolution-Trashing Old Rules to Forge New Ones. Cell (2014) 157:77. 10.1016/j.cell.2014.03.008 24679528

[9] Mercer TR Dinger ME Mattick JS. Long Non-Coding RNAs: Insights Into Functions. Nat Rev Genet (2009) 10:155. 10.1038/nrg2521 19188922

[10] Wilusz JE Sunwoo H Spector DL. Long Noncoding RNAs: Functional Surprises From the RNA World. Genes Dev (2009) 23:1494. 10.1101/gad.1800909 19571179 PMC3152381

[11] Wang KC Chang HY. Molecular Mechanisms of Long Noncoding RNAs. Mol Cell (2011) 43:904. 10.1016/j.molcel.2011.08.018 21925379 PMC3199020

[12] Yuan JH Yang F Wang F Ma JZ Guo YJ Tao QF. A Long Noncoding RNA Activated by TGF-beta Promotes the Invasion-Metastasis Cascade in Hepatocellular Carcinoma. Cancer Cell (2014) 25:666. 10.1016/j.ccr.2014.03.010 24768205

[13] Li D Liu X Zhou J Hu J Zhang D Liu J. Long Noncoding RNA HULC Modulates the Phosphorylation of YB-1 Through Serving as a Scaffold of Extracellular Signal-Regulated Kinase and YB-1 to Enhance Hepatocarcinogenesis. Hepatology (2017) 65:1612. 10.1002/hep.29010 28027578

[14] Fan J Xing Y Wen X Jia R Ni H He J. Long Non-Coding RNA ROR Decoys Gene-Specific Histone Methylation to Promote Tumorigenesis. Genome Biol (2015) 16:139. 10.1186/s13059-015-0852-5 26169368 PMC4499915

[15] Orom UA Shiekhattar R. Long Noncoding RNAs Usher in a New Era in the Biology of Enhancers. Cell (2013) 154:1190. 10.1016/j.cell.2013.08.028 24034243 PMC4108076

[16] Karreth FA Pandolfi PP. ceRNA Cross-Talk in Cancer: When Ce-Bling Rivalries Go Awry. Cancer Discov (2013) 3:1113. 10.1158/2159-8290.CD-13-0202 24072616 PMC3801300

[17] Zhang CL Zhu KP Ma XL. Antisense Lncrna FOXC2-AS1 Promotes Doxorubicin Resistance in Osteosarcoma by Increasing the Expression of FOXC2. Cancer Lett (2017) 396:66. 10.1016/j.canlet.2017.03.018 28323030

[18] Song J Wu X Liu F Li M Sun Y Wang Y. Long Non-Coding RNA PVT1 Promotes Glycolysis and Tumor Progression by Regulating miR-497/HK2 Axis in Osteosarcoma. Biochem Biophys Res Commun (2017) 490:217. 10.1016/j.bbrc.2017.06.024 28602700

[19] Zhang W Zhuang N Liu X He L He Y Mahinthichaichan P. The Metabolic Regulator Lamtor5 Suppresses Inflammatory Signaling Via Regulating Mtor-Mediated TLR4 Degradation. Cell Mol Immunol (2020) 17:1063. 10.1038/s41423-019-0281-6 31467416 PMC7608472

[20] Furukawa M Xiong Y. BTB Protein Keap1 Targets Antioxidant Transcription Factor Nrf2 for Ubiquitination by the Cullin 3-Roc1 Ligase. Mol Cell Biol (2005) 25:162. 10.1128/MCB.25.1.162-171.2005 15601839 PMC538799

[21] Xu S Gong Y Yin Y Xing H Zhang N. The Multiple Function of Long Noncoding RNAs in Osteosarcoma Progression, Drug Resistance and Prognosis. Biomed Pharmacother (2020) 127:110141. 10.1016/j.biopha.2020.110141 32334375

[22] Han J Shen X. Long Noncoding RNAs in Osteosarcoma Via Various Signaling Pathways. J Clin Lab Anal (2020) 34:e23317. 10.1002/jcla.23317 32249459 PMC7307344

[23] Pan K Xie Y. Lncrna FOXC2-AS1 Enhances FOXC2 mRNA Stability to Promote Colorectal Cancer Progression Via Activation of Ca(2+)-FAK Signal Pathway. Cell Death Dis (2020) 11:434. 10.1038/s41419-020-2633-7 32513911 PMC7280533

[24] Guo W Lv P Liu S Xu F Guo Y Shen S. Aberrant Methylation-Mediated Downregulation of Long Noncoding RNA C5orf66-AS1 Promotes the Development of Gastric Cardia Adenocarcinoma. Mol Carcinogen (2018) 57:854. 10.1002/mc.22806 29566283

[25] Bishop MW Janeway KA Gorlick R. Future Directions in the Treatment of Osteosarcoma. Curr Opin Pediatr (2016) 28:26. 10.1097/MOP.0000000000000298 26626558 PMC4761449

[26] Isakoff MS Bielack SS Meltzer P Gorlick R. Osteosarcoma: Current Treatment and a Collaborative Pathway to Success. J Clin Oncol (2015) 33:3029. 10.1200/JCO.2014.59.4895 26304877 PMC4979196

[27] Cong M Li J Jing R Li Z. Long Non-Coding RNA Tumor Suppressor Candidate 7 Functions as a Tumor Suppressor and Inhibits Proliferation in Osteosarcoma. Tumour Biol: J Int Soc Oncodevelopmental Biol Med (2016) 37:9441. 10.1007/s13277-015-4414-y 26781978

[28] Zhang CL Zhu KP Shen GQ Zhu ZS. A Long Non-Coding RNA Contributes to Doxorubicin Resistance of Osteosarcoma. Tumour Biol J Int Soc Oncodevelopmental Biol Med (2016) 37:2737. 10.1007/s13277-015-4130-7 26408180

[29] Jacob J Favicchio R Karimian N Mehrabi M Harding V Castellano L. LMTK3 Escapes Tumour Suppressor miRNAs Via Sequestration of DDX5. Cancer Lett (2016) 372:137. 10.1016/j.canlet.2015.12.026 26739063

[30] Pu Y Zhao F Wang H Cai W Gao J Li Y. MiR-34a-5p Promotes the Multi-Drug Resistance of Osteosarcoma by Targeting the CD117 Gene. Oncotarget (2016). 10.18632/oncotarget.8546 PMC5053736 27056900

[31] Shiraishi M Hayatsu H. High-Speed Conversion of Cytosine to Uracil in Bisulfite Genomic Sequencing Analysis of DNA Methylation. DNA Res An Int J Rapid Publ Rep Genes Genomes (2004) 11:409. 10.1093/dnares/11.6.409 15871463

[32] Lewin J Schmitt AO Adorjan P Hildmann T Piepenbrock C. Quantitative DNA Methylation Analysis Based on Four-Dye Trace Data From Direct Sequencing of PCR Amplificates. Bioinformatics (2004) 20:3005. 10.1093/bioinformatics/bth346 15247106

[33] Hayatsu H Negishi K Shiraishi M. Accelerated Bisulfite-Deamination of Cytosine in the Genomic Sequencing Procedure for DNA Methylation Analysis. Nucleic Acids Symp Ser (Oxf) (2004) 261. 10.1093/nass/48.1.261 17150578

[34] Jyoti K Manjula G Ganachari MS. Application of KW-ANOVA Statistics to Generate Evidence for Cytotoxic Drug Wastage Induced Financial Burden Among Cancer Patients: A Clinical Pharmacist Observation. J Oncol Pharm Pract (2020) 6(7):1559–65. 10.1177/1078155219898710 31948346

[35] Li B Lu W Qu J Zhang Y Wan X. DICER1 Regulates Endometrial Carcinoma Invasion Via Histone Acetylation and Methylation. J Cancer (2017) 8:933. 10.7150/jca.17435 28529604 PMC5436244

[36] Hast BE. Proteomic Analysis of Ubiquitin Ligase KEAP1 Reveals Associated Proteins That Inhibit NRF2 Ubiquitination. Cancer Res (2013) 73:2199. 10.1158/0008-5472.CAN-12-4400 23382044 PMC3618590

[37] Deng H Lv L Li Y Zhang C Meng F Pu Y. miR-193a-3p Regulates the Multi-Drug Resistance of Bladder Cancer by Targeting the LOXL4 Gene and the Oxidative Stress Pathway. Mol Cancer (2014) 13:234. 10.1186/1476-4598-13-234 25311867 PMC4200202

[38] Kim-Wanner SZ Assenov Y Nair MB Weichenhan D Benner A Becker N. Genome-Wide DNA Methylation Profiling in Early Stage I Lung Adenocarcinoma Reveals Predictive Aberrant Methylation in the Promoter Region of the Long Non-Coding RNA PLUT - An Exploratory Study. J Thoracic Oncol (2020). 10.1016/j.jtho.2020.03.023 32272161

[39] Kang KA Hyun JW. Oxidative Stress, Nrf2, and Epigenetic Modification Contribute to Anticancer Drug Resistance. Toxicol Res (2017) 33:1. 10.5487/TR.2017.33.1.001 28133507 PMC5266370

[40] Guo X Xu Y Wang Z Wu Y Chen J Wang G. A Linc1405/Eomes Complex Promotes Cardiac Mesoderm Specification and Cardiogenesis. Cell Stem Cell (2018) 22(6):893–908.e6. 10.1016/j.stem.2018.04.013 29754779

[41] Huang L Zeng L Chu J Xu P Lv M Xu J. Chemoresistancerelated Long Noncoding RNA Expression Profiles in Human Breast Cancer Cells. Mol Med Rep (2018) 18(1):243–53. 10.3892/mmr.2018.8942 PMC6059676 29749447

[42] Cho SW Xu J Sun R Mumbach MR Carter AC Chen YG. Promoter of Lncrna Gene Pvt1 Is a Tumor-Suppressor DNA Boundary Element. Cell (2018) 173:1398. 10.1016/j.cell.2018.03.068 29731168 PMC5984165

[43] Gu Z Hou Z Zheng L Wang X Wu L Zhang C. Lncrna DICER1-AS1 Promotes the Proliferation, Invasion and Autophagy of Osteosarcoma Cells Via Mir-30b/ATG5. Biomed Pharmacother (2018) 104:110. 10.1016/j.biopha.2018.04.193 29772430

[44] Hu XH Dai J Shang HL Zhao ZX Hao YD. High Levels of Long Non-Coding RNA Dicer1-AS1 Are Associated With Poor Clinical Prognosis in Patients With Osteosarcoma. Eur Rev Med Pharmacol Sci (2018) 22:7640. 30536305 10.26355/eurrev_201811_16379

[45] Jin S Antinore MJ Lung FD Dong X Zhao H Fan F. The GADD45 Inhibition of Cdc2 Kinase Correlates With GADD45-mediated Growth Suppression. J Biol Chem (2000) 275:16602. 10.1074/jbc.M000284200 10747892

[46] Hildesheim J Bulavin DV Anver MR Alvord WG Hollander MC Vardanian L. Gadd45a Protects Against UV Irradiation-Induced Skin Tumors, and Promotes Apoptosis and Stress Signaling Via MAPK and P53. Cancer Res (2002) 62:7305. 12499274

[47] Yang F Zhang W Li D Zhan Q. Gadd45a Suppresses Tumor Angiogenesis Via Inhibition of the mTOR/STAT3 Protein Pathway. J Biol Chem (2013) 288:6552. 10.1074/jbc.M112.418335 23329839 PMC3585088

[48] Li Z Gu TP Weber AR Shen JZ Li BZ Xie ZG. Gadd45a Promotes DNA Demethylation Through TDG. Nucleic Acids Res (2015) 43:3986. 10.1093/nar/gkv283 25845601 PMC4417182

[49] Barreto G Schafer A Marhold J Stach D Swaminathan SK Handa V. Gadd45a Promotes Epigenetic Gene Activation by Repair-Mediated DNA Demethylation. Nature (2007) 445:671. 10.1038/nature05515 17268471

[50] Zhou L Wang W Yang C Zeng T Hu M Wang X. Gadd45a Promotes Active DNA Demethylation of the MMP-9 Promoter Via Base Excision Repair Pathway in AGEs-Treated Keratinocytes and in Diabetic Male Rat Skin. Endocrinology (2018) 159:1172. 10.1210/en.2017-00686 29244109

[51] Khalil HS Goltsov A Langdon SP Harrison DJ Bown J Deeni Y. Quantitative Analysis of NRF2 Pathway Reveals Key Elements of the Regulatory Circuits Underlying Antioxidant Response and Proliferation of Ovarian Cancer Cells. J Biotechnol (2015) 202:12. 10.1016/j.jbiotec.2014.09.027 25449014

