# Supplementary material for: Long Noncoding RNA DICER1-AS1 Functions in Methylation Regulation on the Multi-Drugresistance of Osteosarcoma Cells via miR-34a-5p and GADD45A
Source: Front Oncol. 2021 Jul 9;11:685881. doi: 10.3389/fonc.2021.685881 (PMC8299526; doi:10.3389/fonc.2021.685881)

Long noncoding RNA DICER1-AS1 functions in methylation regulation on the multi-drugresistance of osteosarcoma cells via miR-34a-5p and GADD45A

Supplementary Fig. 1

The sequence of primers and probes of DICER1-AS1：
hDICER1-AS1 F: 5′-TGGGATTACGGGCGTGAG-3′
hDICER1-AS1 R: 5′-CCTGGGCACTCCTTCAGC-3′
hDICER1-AS1 P: 5′-CY5-CACCGTACCCAGCCTGCTTCCTG-3′

The sequence of primers and probes of GADD45A：
hGADD45A F: 5′-CCCCGATAACGTGGTGTTGT-3′
hGADD45A R: 5′-GATGTCGTTCTCGCAGCAAA-3′
hGADD45A P: 5′-FAM-CTCTGCAGATCCACTTCACCCTGATCCA-3′

The sequence of si-DICER1-AS1：
si-DICER1-AS1_001 GGAATAACTTCCAACAAGT
si-DICER1-AS1_002 GACGATTGTCTAAGGATGA
si-DICER1-AS1_003 CACATTTCCTTACTCCATA

The sequence of si-GADD45A：
si-GADD45A-1
GCGAGAACGACAUCAACAUTT
AUGUUGAUGUCGUUCUCGCTT
si-GADD45A-2
CCUGCCUUAAGUCAACUUATT
UAAGUUGACUUAAGGCAGGTT
si-GADD45A-3
CCGAAAGGGUUAAUCAUAUTT
AUAUGAUUAACCCUUCCCGGTT

The sequence of sh-DICER1-AS1：
DICER1-AS1-shRNA(GGAATAACTTCCAACAAGT)
TATGTTTTAAAATGGACTATCATATGCTTACCGTAACTTGAAAGTATTTCGAT
TTCTTGGGTTTATATATCTTGTGGAAAGGACGCGGGATCCCGGAATAACTTC
CAACAAGTTTGATATCCGACTTGTTGGAAGTTATTCCTTTTTTCCAAAAGCT
TAAGTTTAAACCGCTGATCAGCCTCGACTGTGCCTCTAAATAGTATCATTGG
GC

The sequence of hsa-miR-34a-5p inhibitor：
ACAACCAGCTAAGACACTGCCACGATACAACCAGCTAAGACACTGCCATC
ACACAACCAGCTAAGACACTGCCA

The sequence of hsa-miR-34a-5p-O/E：
TGGCAGTGTCTTAGCTGGTTGT

The sequence of hsa-miR-34a-5p NC-O/E：
TTCTCCGAACGTGTCACGT

The sequence of DICER1-AS1-O/E：
ATGTTGGCCATGATGGTCTCGATTTCTGACCTTGTCATCCGTCCGCCTTGG
CCTCCCAAAGTGCTGGGATTACGGGCGTGAGCCACCGTACCCAGCCTGCT
TCCTGTTTTAACAGACGAAGAAATGGAATAACTTCCAACAAGTTGGAGCT
GAAGGAGTGCCCAGGCTGCGGATGGAGGAAGGACGTGCTCGGGAGAAGG
TGAAGATGGGAGAGAAGCAAGATCTTCTGTGTTTGGACCAAGGACACATT
TCCTTACTCCATAGCAGCTGTGGTCAGTGGCTTAGCTCGGACAAGGAGAT
GAGAGCCCATGTGTTGTGAGGGTTCTTCTGGGAACTCTGAGAAGGCAGGA
GCCGCCCCCGCCCTTCACTGCCTCTCTTCCATTCTGCAGCATGGAATATGG
ATGTGTGGTCTGGATGTACAGACACCATTTTGGACCATAAAGACAAGGAC
GATTGTCTAAGGATGACACAGCCAGAGCACTGAGAGTGGCGAGAAGGAC
AGGGGAGCTGCTGTGCCAAGCAGGACTGTCTCTCTTAAGAGAAAAATAA

The sequence of DICER1-AS1-BSP primers:
DICER1-AS1-F 5' TATTTGGGTTTGTAGTAGT 3'
DICER1-AS1-R 5' AAACCAATAAAATTCTCCA 3'

The sequence of DICER1-AS1-wt：
CTCGAGCGGATGGAGGAAGGACGTGCTCGGGAGAAGGTGAAGATGGGAG
AGAAGCAAGATCTTCTGTGTTTGGACCAAGGACACATTTCCTTACTCCATA
GCAGCTGTGGTCAGTGGCTTAGCTCGGACAAGGAGATGAGAGCCCATGTG
TTGTGAGGGTTCTTCTGGGAACTCTGAGAAGGCAGGAGCCGCCCCCGCCC
TTCACTGCCTCTCTTCCATTCTGCAGCATGGAATATGGATGTGTGGTCTGGA
TGTACAGACACCATTTTGGACCATAAAGACAAGGACGATTGTCTAAGGATG
ACACAGCCAGAGCACTGAGAGTGGCGAGAAGGACAGGGGAGCTGCTGTG
CCAAGCAGGACTGTCTCTCTTAAGAGGCGGCCGC

The sequence of DICER1-AS1-mut：
CTCGAGCGGATGGAGGAAGGACGTGCTCGGGAGAAGGTGAAGATGGGAG
AGAAGCAAGATCTTCTGTGTTTGGACCAAGGACACATTTCCTTACTCCATA
GCAGCTGTGGTCAGTGGCTTAGCTCGGACAAGGAGATGAGAGCCCATGTG
TTGTGAGGGTTCTTCTGGGAACTCTGAGAAGGCAGGAGCCGCCCCCGCCC
TTGAGTTCATCTCTTCCATTCTGCAGCATGGAATATGGATGTGTGGTCTGGA
TGTACAGACACCATTTTGGACCATAAAGACAAGGACGATTGTCTAAGGATG
ACACAGCCAGAGCACTGAGAGTGGCGAGAAGGACAGGGGAGCTGCTGTG
CCAAGCAGGACTGTCTCTCTTAAGAGGCGGCCGC

Supplementary Fig. 2

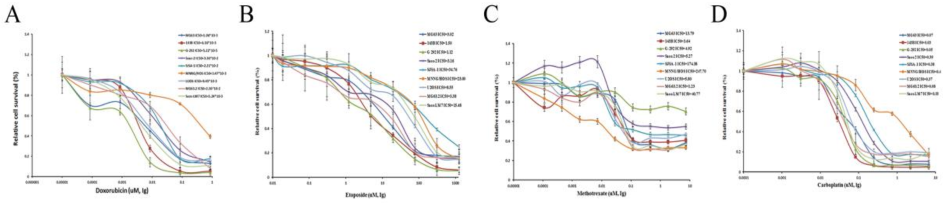

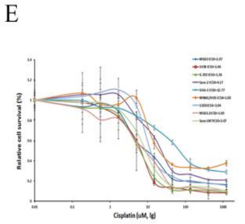

F

Relative IC50	143B	G-292	MG63.2	MG63	SaOS.2	U2OS	SaOS-LM7	MNNG/HOS	SJSA-1
Doxorubicin	1.00	1.00	90.80	9.60	8.77	10.11	19.28	39.77	33.87
Etoposide	1.34	1.00	3.02	2.69	2.82	7.64	13.39	20.53	31.03
Methotrexate	2.96	4.00	1.00	11.21	4.28	4.71	33.15	201.38	141.77
Cisplatin	1.46	1.36	1.00	2.07	4.17	3.04	3.07	1.00	12.77
Carboplatin	1.00	1.75	2.46	2.35	12.77	12.18	10.67	7.84	12.21
Chemoresistance Index	1.00	1.17	12.68	3.60	4.23	4.86	10.26	22.00	29.89

Drug resistance profiling of nine osteosarcoma cell lines.

(**A**-**E**).IC_{50} values of the five indicated chemotherapeutics for nine osteosarcoma cell lines. The cell survival rates were calculated as percentages relative to the mock treatment and plotted against lg μM of drug. (**F**).The IC_{50} (-fold) values relative to those of the most sensitive cell line (143B) are presented in the table. Judged by the fold difference over the lowest IC_{50}, in this manuscrpt, G-292 and 143B regard as the multi-drugsensitive cell lines, while SJSA-1 and MNNG/HOS as the resistant cell line.

Supplementary Fig. 3

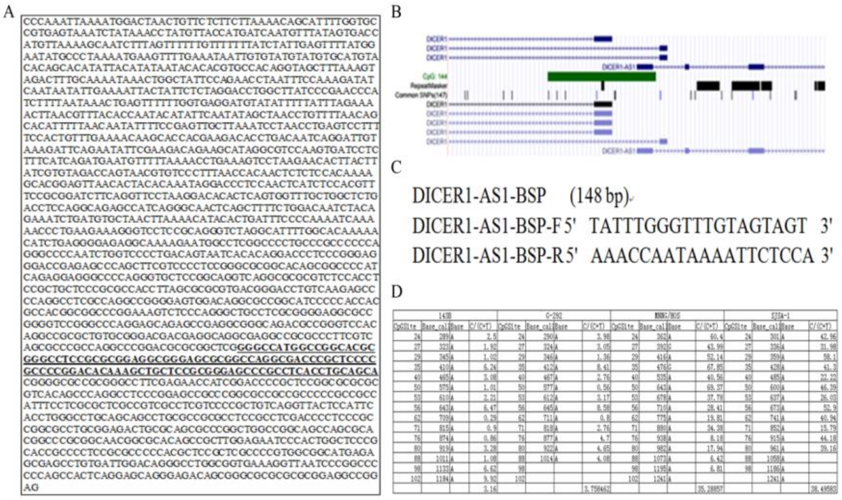

The promoter region, CG sites, BSP primers and the results of sequence of each site in four OS cell lines. (**A**).The 2000bp promoter region of DICER1-AS1, the PCR amplification sequence riched in CG island was marked with bold and underline.

(**B**).The information of DICER1-AS1 in UCSC (http://genome.ucsc.edu/). (**C**).The BSP primers of DICER1-AS1. (**D**).The results of each site in four OS cell lines.

Supplementary Fig. 4

The full-length gels of the Fig 3E and Fig 3F western analyses used in the revised manuscript.

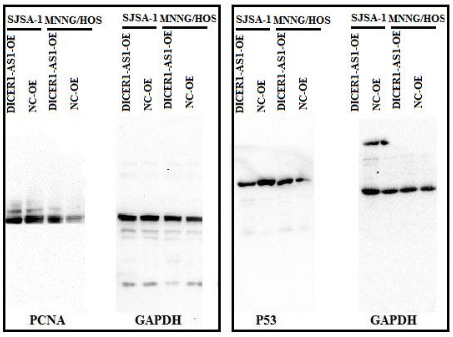

The full-length gels of the Fig 4C western analyses used in the revised manuscript.

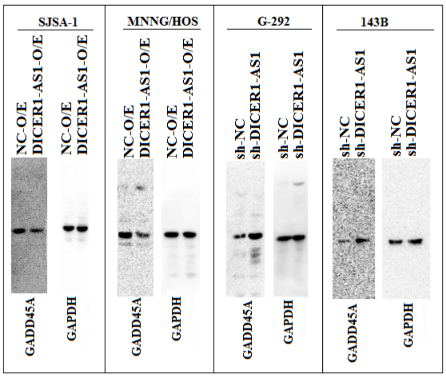

The full-length gels of the Fig 4D western analyses used in the revised manuscript.

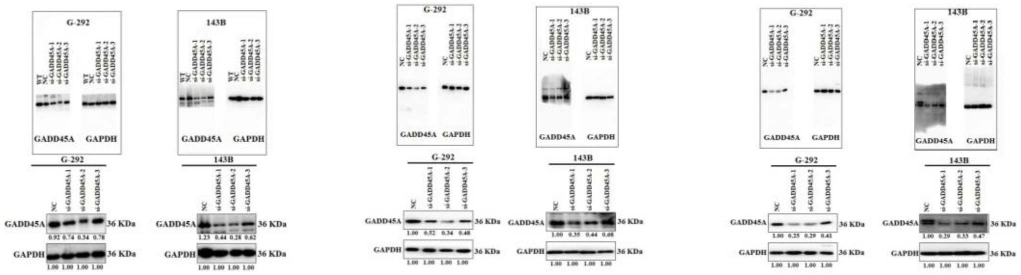

The full-length gels of the Fig 6C and Fig 6D western analyses used in the revised manuscript.

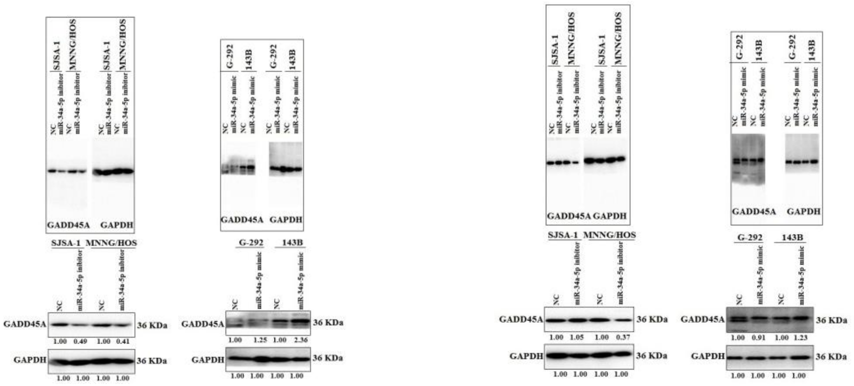

Supplementary Fig. 5

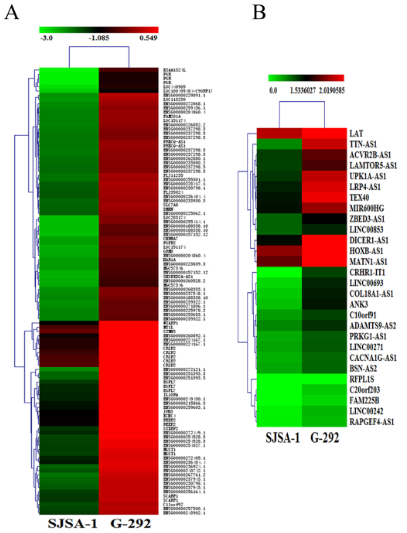

(**A**).Hierarchical clustering analysis of RNA-seq lncRNAs that were differentially expressed between G-292 and SJSA-1 cells, the coordinates on the right represent the expression of the lg(fpkm+1). (**B**).Hierarchical clustering analysis of RNA-seq lncRNAs that negatively correlates with miR-34a-5p, the coordinates on the right represent the expression of the lg(fpkm+1).

Supplementary Fig. 6

Gene ID	Name	Position	Strand	SJSA-1 RPKM	G-292 RPKM	G-292/SJSA-1
ENST00000582491	CRHR1-IT1	chr17:45638975-45646229	+	0.008	0.135	17.290
ENST00000582847	TTN-AS1	chr2:178522827-178620217	+	0.052	0.824	15.787
ENST00000354453	LAT	chr16:28984826-28990783	+	0.763	9.807	12.858
ENST00000432518	LINC00693	chr3:28575278-28758337	+	0.018	0.202	11.527
ENST00000397787	COL18A1-AS1	chr21:45419716-45425070	-	0.021	0.227	10.835
ENST00000414786	ANK3	chr10:60026298-60733526	-	0.024	0.250	10.486
ENST00000602385	TERC	chr3:169764610-169765060	-	164.017	1033.610	6.302
ENST00000439819	DICER1-AS1	chr14:95157688-95179933	+	0.289	1.479	5.115
ENST00000443196	UPK1A-AS1	chr19:35667948-35673291	-	0.164	0.799	4.867
ENST00000502049	LRP4-AS1	chr11:46846411-46874416	+	0.217	0.890	4.110
ENST00000328404	TEX40	chr11:64300391-64304767	+	0.253	0.966	3.813
ENST00000515356	ZBED3-AS1	chr5:77086798-77148351	+	0.089	0.337	3.787
ENST00000441531	ACVR2B-AS1	chr3:38451027-38454820	-	0.144	0.512	3.550
ENST00000392630	C10orf91	chr10:132445210-132448321	+	0.039	0.136	3.458
ENST00000608990	C20orf203	chr20:32631625-32651981	-	0.006	0.021	3.458
ENST00000460833	ADAMTS9-AS2	chr3:64684870-65011468	+	0.052	0.180	3.458
ENST00000598158	LAMTOR5-AS1	chr1:110407809-110416274	+	0.136	0.471	3.458
ENST00000502764	HOXB-AS1	chr17:48544351-48551241	+	0.637	1.951	3.063
ENST00000420193	PRKG1-AS1	chr10:52296848-52314128	-	0.032	0.096	2.997
ENST00000421378	LINC00271	chr6:135497801-135690838	+	0.035	0.102	2.882
ENST00000505495	CACNA1G-AS1	chr17:50556207-50562108	-	0.030	0.082	2.690
ENST00000414282	SNHG7	chr9:136724594-136728184	-	102.005	258.103	2.530
ENST00000421598	BSN-AS2	chr3:49549306-49554366	-	0.033	0.084	2.497
ENST00000449175	MIR600HG	chr9:123109494-123115477	-	0.269	0.671	2.492
ENST00000437615	LINC00242	chr6:169788790-169798825	-	0.012	0.028	2.305
ENST00000429328	LINC00853	chr1:47179250-47180339	+	0.088	0.202	2.305
ENST00000423546	FAM225B	chr9:113104723-113111677	-	0.007	0.017	2.305
ENST00000461286	RFPL1S	chr22:29437015-29442129	-	0.003	0.007	2.305
ENST00000414532	MATN1-AS1	chr1:30718772-30726746	+	0.524	1.207	2.305
ENST00000435328	RAPGEF4-AS1	chr2:172723189-172736206	-	0.013	0.025	2.017

Using pathway enrichment with Kegg data base to screen the lncRNA genes (the ratio>2) related to miR-34a-5p, which is our previous focused.

Supplementary Fig. 7

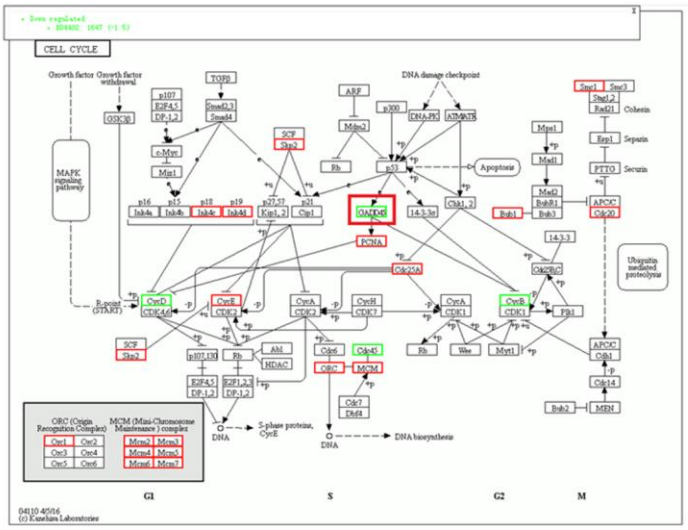

The associated genes analysis of GADD45A in cells by pathway enrichment with Kegg data base.

Supplementary Fig. 8

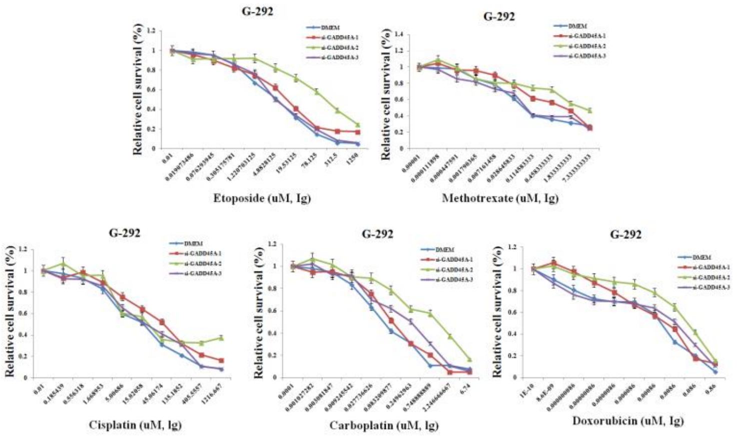

G-292 cells transfected 12hours with the three siRNA of GADD45A were seeded in triplicate in 96-well plates at a density of 0.5×10^4 cells/well and treated with 4-fold serially diluted drugs concentration gradient for another 72 hours, then, cell viability was measured using CCK-8.

Supplementary Fig. 9

Gene Ontology enrichment analyses with differentially expressed gene

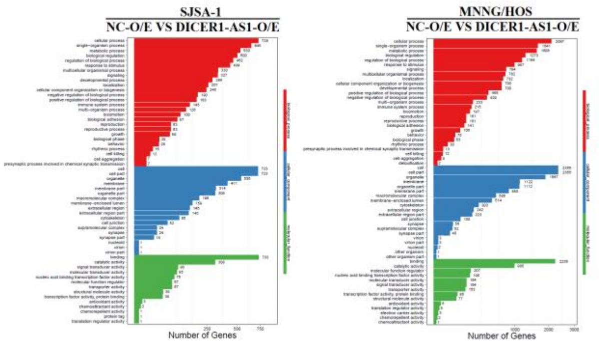

Supplementary Fig. 10

The snapshots of RNA-seq in GADD45A loci

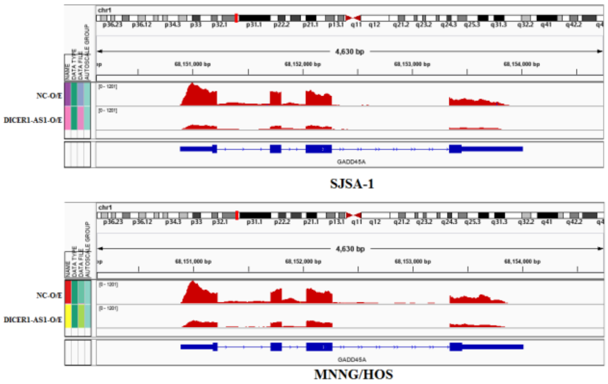

Supplement: Supplementary file 1 [file DataSheet_1.pdf]
